# Supplementary material for: Exploring team dynamics and performance in extended space missions using agent-based modeling
Source: PLoS One. 2025 Oct 8;20(10):e0332496. doi: 10.1371/journal.pone.0332496 (PMC12507196; doi:10.1371/journal.pone.0332496)
Supplement: Attachment — Submitted filename: Response to Reviewers_PLOSONE.pdf [file pone.0332496.s001.pdf]

Dear Editors and Reviewers,

We would like to extend our sincere gratitude for the detailed and insightful feedback provided during the review of our manuscript. The comments and suggestions offered have greatly assisted us in clarifying our theoretical foundations, refining our methodological approach, and improving the overall presentation of our study.

In this revision, we have thoroughly addressed each of the reviewers' comments individually. Our responses to specific reviewer comments are provided clearly in green text, and corresponding modifications within the revised manuscript have been highlighted in blue for easy reference.

We believe that these improvements strengthen both the academic rigor and clarity of our manuscript and align closely with the reviewers' valuable recommendations.

Thank you again for your careful consideration of our work. We look forward to your feedback on this revised version.

Sincerely,
Iser Pena, Stevens Institute of Technology
Hao Chen, Stevens Institute of Technology

Reviewer 1

Thank you for the opportunity to review Team Dynamics and Performance in Extended Space Missions Using Agent-Based Modeling. I was excited to read this manuscript and applaud the authors for attending to critical team dynamic issues related to extended space travel. The use of agent-based modeling to address the topic was commendable and, as the authors alluded to, offers to advance our understanding of psychological issues relevant to future space travel. While I applaud the general premise of the research conducted for the paper, I have some concerns about the theorizing and presentation of the work.

> We sincerely appreciate reviewer's thoughtful and constructive feedback and are pleased to hear the recognition of the importance of exploring team dynamics through agent-based modeling for extended space missions. We acknowledge the reviewer's concerns regarding the theoretical framing and presentation of our study. In response, we have revisited our theoretical foundations and enhanced the manuscript by clarifying and refining the connections between the psychological theories employed and our modeling approach. Additionally, we have restructured parts of the manuscript to improve clarity and coherence, ensuring a more seamless flow of ideas. We believe these revisions significantly strengthen the theoretical rigor and overall readability of the paper.

Comments:

1. The authors used a 'gap argument' to make the case for the motivation for this research (for example: "…current research lacks comprehensive models…"). Instead of pointing out the gap in the current literature, I recommend that the authors make a stronger case for the unique contribution of this research. The absence of more extensive research on team dynamics in extended space travel is likely due to a valid reason, so highlighting why this research is relevant now will help the authors highlight how they are paving the way for future studies.

   > As suggested by the reviewer, we highlighted the relevance of the research and follow the advice as follows:
   >
   > Page 2: "With NASA's Artemis missions now underway and crewed Mars missions becoming increasingly concrete, there is a timely and critical need to develop predictive tools capable of assessing and optimizing team composition, psychological resilience, and operational effectiveness under realistic, Mars-like conditions. Existing studies have primarily relied on shorter-duration missions or terrestrial analog environments, such as the ISS (International Space Station), Mir, Mars-500, and HI-SEAS (Hawai'i Space Exploration Analog and Simulation), which do not fully capture the cumulative psychological and operational complexities of multi-year Mars expeditions [2, 3]. Our study addresses this critical gap by explicitly modeling how psychological adaptation interacts with individual personality traits over long-duration missions. Rather than focusing solely on trait-based outcomes or environmental triggers, we adopt a systems perspective that captures their dynamic interplay across time. This contribution supports the development of targeted interventions for crew selection, cohesion training, and resilience-building protocols essential to the success of future deep-space exploration."

2. Psychological adaptation is introduced in the literature review; however, the introduction was abrupt as this construct was not previously mentioned. I see how psychological adaptation

could be relevant. Still, I encourage the authors to consider how adaptation fits with the narrative about the role of the Big Five dimensions and to frame the opening of the paper with this argument to make it clear why they refer to this literature.

> We agree with the observation and we added a paragraph regarding the FFM as follows:
>
> Page 2: "As mission durations extend into deep space, sustaining crew performance requires more than technical skill, it hinges on psychological adaptation, the dynamic process through which individuals adjust their thoughts, behaviors, and emotions in response to prolonged isolation, operational stress, and limited social interaction. This adaptive capacity is not random, it is strongly shaped by stable personality traits, particularly those outlined in the FFM. These traits influence how individuals respond to stress, maintain motivation, and interact within a small, confined team over time. Framing psychological adaptation as an expression of personality under chronic stress provides a more nuanced understanding of astronaut behavior, enabling us to anticipate how different personality compositions may influence team functioning. Integrating this perspective into selection, training, and mission planning, space agencies can proactively support resilience, cohesion, and long-term performance in the extreme conditions of Mars-class missions."

3. What makes personality a key factor especially important in high-stress and isolated environments? The authors make this statement without explaining. I don't disagree with the statement, but it would be helpful to explain the reasoning behind it—which would also strengthen the rationale for the paper.

> Thank you for bringing this to our attention. Explanation is being expanded as below:
>
> Page 5: "These traits fundamentally shape how individuals perceive and respond to prolonged stress, confinement, and interpersonal friction conditions that cannot be fully mitigated through training alone. In such extreme environments, where support from Earth is delayed and crews must operate autonomously, personality-driven behaviors like emotional regulation, adaptability, and social sensitivity become critical for maintaining cohesion and operational stability [8, 9]. "
>
> Page 5: "It enables mission planners to anticipate stress responses, communication dynamics, and interpersonal compatibility, offering predictive value for selecting and supporting crews capable of sustaining long-duration missions [10]."

4. I encourage the authors to review the following to help bolster their discussion of the role of team composition in the context of space missions. While this paper is cited, there is more from this work, which primarily covers team composition— directly related to the current manuscript. o Bell, S. T., Brown, S. G., Outland, N. B., & Abben, D. R. (2015). Critical team composition issues for long-distance and long-duration space exploration: A literature review, an operational assessment, and recommendations for practice and research.

> Based on the reviewer's recommendation, we expand on Bell et al.'s work below:
>
> Page 3: "...particularly given the extreme psychological and operational demands of isolated, confined, and autonomous environments. Bell et al. [1] provide one of the most

comprehensive assessments of this topic, emphasizing that team composition is not only about individual competence but also about the configuration of interpersonal compatibility that influences both social integration and team performance. Their review identifies two major pathways through which composition affects mission outcomes: (1) by shaping the quality of interpersonal relations such as cohesion, conflict, and subgroup formation and (2) by affecting critical team processes such as coordination, shared cognition, and adaptability. The report also highlights that both surface-level (e.g., age, sex, culture) and deep level attributes (e.g., personality traits, values, dominance) can lead to dysfunction when not aligned or managed, particularly in environments requiring high interdependence and limited external support. These insights underscore the importance of proactive team design and compatibility assessments in spaceflight planning. "

5. In the description of the model, the heterogeneous teams varied not just by personality but also by cultural background, roles, and skills. If we see effects in the heterogeneous teams that do not replicate in the homogeneous teams, how can we be confident that the differences are due to personality? From an experimental perspective, varying cultural backgrounds, roles, and skills for the heterogeneous teams only would introduce a confound. Could you explain the decision to vary other characteristics besides personality for the heterogeneous teams only?

We appreciate the reviewer's important comment regarding potential confounding variables. In response, we revised the manuscript to clarify that only personality traits and skill/role assignments vary across experimental conditions. We added a dedicated Section 2.3 titled "Team Composition Conditions" (Page 11), which describes our 2×2 experimental design.

We also updated the Results section to reflect this revised factorial structure, with new figures and analyses distinguishing outcomes across all four composition scenarios.

Page 11: "To investigate how personality traits and role specialization influence team performance and psychological adaptation during long-duration missions, we employ a 2×2 experimental design (Table 2). This framework crosses two levels of personality heterogeneity (low vs. high) with two levels of skill/role heterogeneity (low vs. high), resulting in four distinct team composition conditions. Each condition represents a different configuration of individual differences and functional diversity within the crew, allowing us to compare outcomes across varying degrees of team complexity."

A new table titled **"2×2 Factorial Design of Team Composition: Varying Personality and Skill/Role Heterogeneity"** (Table 2, Page 12) illustrates the four simulation conditions: (A) Low P / Low R, (B) Low P / High R, (C) High P / Low R, and (D) High P / High R.

The assignment of skills and roles is operationalized during the initialization of astronaut agents. In the low skill/role heterogeneity conditions, all agents are assigned the same functional role (e.g., engineer) and identical task proficiency values. In contrast, high skill/role heterogeneity conditions assign each astronaut a distinct role such as pilot, engineer, medic, or scientist with role specific proficiencies.

While the model does not simulate explicit interdependence between roles, the variation in assigned responsibilities introduces differences in how agents contribute to mission tasks. For instance, a medic agent may be more effective in responding to health-related events, while an engineer may better handle equipment failures.

For clarity, we refer to the four configurations using the following shorthand: (A) Homo – Skill + Personality, (B) Hetero – Skill, (C) Hetero – Personality, and (D) Hetero – Skill + Personality. These labels are used consistently throughout the manuscript.

6. Team cohesion is introduced as an outcome very late in the paper. It would be beneficial to introduce cohesion earlier in the paper and discuss its theoretical fit, rather than it appearing as an afterthought.

We appreciate the reviewer's comment and agree that cohesion plays a foundational role in linking team composition with long-term mission functioning. In response, we revised the literature review section to introduce cohesion earlier in the manuscript and clarify its theoretical relevance

Page 3: "Cohesion defined as the strength of social bonds and shared commitment among crew members has emerged as a core factor in mission stability. Rather than a static trait, it evolves over time and is shaped by interpersonal compatibility, communication style, and group adaptation. Analog missions such as Mars-500 and HI-SEAS have shown that cohesion tends to decline or fluctuate during periods of stress, especially mid-mission [11, 12]. These cohesion patterns reflect how interpersonal dynamics adapt under isolation and stress, emphasizing that team composition directly influences the crew's psychological resilience [1]."

7. Overall, the theoretical framework could be more focused. By honing in on the precise contribution of the study, the authors can more strongly communicate the potential impact of their research. Once the focus is clarified, the authors should revisit the literature review to align the opening with the methodology.

   To reiterate, the precise focus of the study is a bit unclear. However, with some adjustments, the paper could benefit from a coherent focus and stronger theorizing about the role of crew personality composition in the context of extended space missions.

   I hope the authors find my comments helpful in strengthening their paper and the overall contribution of their research.

We thank the reviewer for the thoughtful feedback. We refined the focus of the paper and revised the introduction and literature review to align with the study's core contribution.

Reviewer 2

Thank you for the opportunity to review your paper entitled "Exploring Team Dynamics and Performance in Extended Space Missions Using Agent-Based Modeling." This paper presented an agent-based model that simulated personality composition in teams and the impact of different personality configurations on important outcomes, such as stress and performance, during a long-duration space flight. I was impressed by both the presentation and execution of this work, and I found this paper to be well-written and highly transparent (especially regarding modeling decisions). Below, I include several considerations that I hope will have a positive impact on this line of research.

We sincerely thank the reviewer for the encouraging and detailed feedback. We are especially grateful for the recognition of our modeling transparency and the clarity of our framework. Your comments reinforce the importance of integrating psychological realism into simulations of long-

duration missions. We have incorporated your suggestions to further strengthen the manuscript and expand on areas of potential refinement, as detailed in our specific point-by-point responses below.

Comments:

1. In your literature review, you mention several studies that have simulated long-duration space flight via computational modeling. I think it would also be beneficial to include recent work by Alina Lungeanu, as she and her collaborators have discussed and published several computational models simulating teams in long-duration space flight (e.g., Lungeanu et al., 2022; Antone et al., 2023; Mesmer-Magnus et al., 2020). This may help to also bring in work from communications and the organizational sciences into your literature review so your work can speak to a wider audience.

   Thank you for your thoughtful suggestions. We have incorporated the recommended studies into our literature review as shown below:

   Page 4: "Recent contributions from organizational and communication sciences have emphasized the importance of conceptualizing spaceflight teams not merely as cohesive units, but as adaptive systems embedded within evolving task environments. Lungeanu et al. [5] adopt a task management perspective to analyze multiteam systems in spaceflight, revealing how coordination patterns shift under conditions of increasing crew autonomy, temporal pressure, and dynamic operational priorities. Their findings underscore the need to understand how team structures evolve as astronauts assume responsibilities traditionally managed by Earth-based support. Building on this, Antone et al. [4] highlight the value of computational approaches to examine how individual differences, such as personality traits, interact with team composition and influence performance over time. They call for integrative frameworks capable of capturing nonlinear, time-sensitive team processes. Similarly, Mesmer-Magnus et al. [6] identify key complexities unique to spaceflight teams including delayed communication, adaptive role shifts, and cumulative psychological stressors and advocate for a new generation of theoretical and modeling tools to guide crew design and intervention strategies. Together, these perspectives provide a conceptual foundation for exploring more dynamic and empirically grounded representations of team functioning in spaceflight. "

2. I appreciate the attempt to create a figure that succinctly explains the differences between the real-world and the simulated environment of an ABM in Figure 1, but I'm not sure Figure 1 is really adding anything useful to your manuscript. Because I already understand ABMs, I see what you were trying to do with this figure, but I'm not sure someone who is unfamiliar with ABMs is going to understand what this figure means based on its current form. You might consider either revising it or dropping it from the paper, as comparatively, your other figures provide much more information than this one.

   We appreciate the reviewer's insightful comment and we have removed the figure and merged the ABM introduction to Sec. 1.1.3 ABM for Investigating Team Dynamics and Performance in Page 6

3. On page 8-9, you discuss the IMO model, which is one of the foundational models for team processes. However, in Figure 2 and in the later text on page 9, you lay out your IMO model

as an "IMOI model" instead (i.e., output loops back to impact input again, and the process updates and repeats). In the teams literature, the IMOI model has largely replaced the IMO model because it captures the dynamic nature of teamwork (which is frequently not just one performance episode, but many performance episodes), and you capture this idea already in text and in your figure, but just not in your labeling and citations, so I would recommend switching to the IMOI framework for your paper (see Ilgen et al., 2005).

> We thank the reviewer for pointing this out. We have now switching from IMO to IMOI to capture accurately the idea of our work as suggested.
>
> Page 9: "Figure 1 presents the simulation's conceptual framework, structured according to the Input–Mediator–Output–Input (IMOI) model proposed by Ilgen et al. [7]. This framework extends the traditional Input–Mediator–Output (IMO) model by emphasizing the recursive nature of team dynamics. In the context of long-duration space missions, team outputs such as cohesion, performance, and health do not represent endpoints but instead feed back into the system, influencing subsequent inputs and mediators."
>
> "Fig 1: Input-Mediator-Output-Input (IMOI) conceptual framework for the Agent-Based Model"

4. You define heterogenous teams (in Table 1) as having not only heterogeneity in terms of personality composition, but also in terms of skills/roles and task proficiency. However, in your discussion of your model results, you only mention the findings as resulting from heterogeneity in personality composition, and not the heterogeneity that was built into the model to represent different but overlapping roles and expertise among team members. However, the teams literature would suggest that heterogeneity in terms of roles and expertise is also a critical predictor of important team outcomes such as stress and performance. Are you isolating the effects of heterogeneity of personality from the effects of heterogeneity of skills/roles? If so, how? And if not, this is something that I would recommend revising in order to make sure your model results are due to your intended predictors and not extraneous influences. Perhaps you could have a 2x2 design where you test high (low) personality heterogeneity and high (low) skill/role heterogeneity to parse out the effects of each more completely.

> We appreciate this important comment. We agree that team heterogeneity includes both personality traits and functional roles, and that isolating their effects is necessary for a clearer interpretation of team performance outcomes. In response, we revised our simulation design to implement a 2×2 factorial structure that explicitly tests all combinations of high/low personality heterogeneity and high/low skill/role heterogeneity along with a new section 2.3 Team Composition Conditions.
>
> In addition, the Results section was fully updated to reflect this revised factorial structure, with new figures and analyses distinguishing outcomes across all four composition scenarios.
>
> Page 11: "To investigate how personality traits and role specialization influence team performance and psychological adaptation during long-duration missions, we employ a 2×2 experimental design (Table 2). This framework crosses two levels of personality heterogeneity (low vs. high) with two levels of skill/role heterogeneity (low vs. high), resulting in four distinct team composition conditions. Each condition represents a different configuration of individual differences and functional diversity within the crew,

allowing us to compare outcomes across varying degrees of team complexity."

A new table titled **"2×2 Factorial Design of Team Composition: Varying Personality and Skill/Role Heterogeneity"** (Table 2, Page 12) illustrates the four simulation conditions: (A) Low P / Low R, (B) Low P / High R, (C) High P / Low R, and (D) High P / High R.

The assignment of skills and roles is operationalized during the initialization of astronaut agents. In the low skill/role heterogeneity conditions, all agents are assigned the same functional role (e.g., engineer) and identical task proficiency values. In contrast, high skill/role heterogeneity conditions assign each astronaut a distinct role such as pilot, engineer, medic, or scientist with role specific proficiencies.

While the model does not simulate explicit interdependence between roles, the variation in assigned responsibilities introduces differences in how agents contribute to mission tasks. For instance, a medic agent may be more effective in responding to health-related events, while an engineer may better handle equipment failures.

For clarity, we refer to the four configurations using the following shorthand: (A) Homo – Skill + Personality, (B) Hetero – Skill, (C) Hetero – Personality, and (D) Hetero – Skill + Personality. These labels are used consistently throughout the manuscript.

5. Related to my last comment, how were the overlapping skills sets (i.e., homogenous teams) versus diverse skill sets (i.e., heterogenous teams) operationalized? Perhaps I missed it, but I don't remember seeing this explicitly outlined in your paper. Because it's still possible, in my opinion, for these variables to have important effects in your model, this is something that would benefit from more clarity.

We appreciate the reviewer's insightful comment and agree that clarifying how skill sets were operationalized strengthens the paper. We have now incorporated the following description into the manuscript:

Page 11: "The assignment of skills and roles is operationalized during the initialization of the astronaut agents. In the low skill/role heterogeneity conditions, all agents are assigned the same functional role (e.g., engineer) and given identical task proficiency values. This simulates a homogeneous team in which all members approach mission tasks with the same set of capabilities and responsibilities, minimizing functional diversity. In contrast, the high skill/role heterogeneity conditions assign each astronaut a distinct role such as pilot, engineer, medic, or scientist each accompanied by a unique set of task proficiencies tailored to that role. These proficiencies influence how each agent performs in domain specific tasks embedded within the simulation's daily operational routines.
"While the model does not simulate explicit interdependence or coordination demands between roles, the variation in assigned responsibilities and task strengths introduces differences in how each agent contributes to mission objectives. For instance, a medic agent may be more efficient in responding to health-related events, while an engineer performs may be better in equipment maintenance scenarios. This functional specialization allows us to examine whether performance variability and stress responses emerge from skill/role diversity alone, or whether they are moderated by the interaction between skills and personality traits. "

6. On p. 18–19, you discuss team cohesion within the model. However, some of your language suggests that individual astronauts/agents having individual "cohesion scores" (e.g., "For

instance, astronaut A3 experienced a notable dip in cohesion around day 167...”). Yet, cohesion is a group-level metric and not an individual-level metric, so I would clarify in this section what you mean by an individual's cohesion. I suspect you mean to discuss their social standing with others, rather than their individual cohesion.

> We appreciate the reviewer's comment and agree that team cohesion is conceptually a group-level construct. In our simulation, cohesion is modeled through pairwise relationships among agents, which are then averaged to quantify each astronaut's degree of social integration within the team. The language in our original manuscript may have unintentionally implied that cohesion was treated as an individual trait rather than a relational metric. We have revised the relevant section to clarify this point as below:
>
> Page 20-21: “Although cohesion is conceptually a group-level construct, the model tracks each astronaut's average pairwise cohesion with the rest of the team over time. These values are not individual traits, but reflect each agent's degree of social integration within the group, based on the strength of their reciprocal social ties. This framing enables analysis of how interpersonal connections vary dynamically across agents and scenarios.”

7. Also related to my last comment, how was cohesion operationalized? It sounds like it is a network of social ties between astronauts/agents, but are these ties directed or undirected? Is it meaningful if one astronaut says they have a positive relationship with another, and that person does not reciprocate? Adding additional details here would be helpful to make sure the reader can follow along with the details of what is happening with cohesion.

> We appreciate the reviewer's analysis. To clarify this aspect, we have added an explanation in the cohesion analysis section. The model treats cohesion as a symmetric, undirected construct based on bidirectional interaction values between astronaut pairs. Each pairwise cohesion value is equal in both directions, and non-reciprocal relationships are not separately tracked in this version of the simulation. Described as below;
>
> Page 21: “In this model, cohesion is computed as a symmetric, undirected metric representing the average strength of mutual social ties between astronaut pairs. The cohesion matrix is updated over time based on bidirectional interactions meaning that the cohesion between astronaut A1 and astronaut A2 is always equal to that between A2 and A1. One-sided perceptions or non-reciprocated relationships are not modeled separately. This design reflects the assumption that social integration arises from mutual experiences and sustained exchanges rather than unilateral perceptions.”

8. Overall, I found this paper to be quite an interesting and enjoyable read. I hope you find these comments useful as you continue to develop this work.

> We sincerely thank the reviewer for the thoughtful engagement and encouraging words. We appreciate the constructive feedback and have incorporated revisions throughout the manuscript to strengthen the clarity and contribution of the study.

References

[1] Bell ST, Brown SG, Abben DR, Outland NB. Team composition issues for future space exploration: A review and directions for future research. Aerospace Medicine and Human Performance. 2015;86(6):548–556. `https://doi.org/10.3357/AMHP.4251.2015`.

[2] Salas E, Grossman R, Hughes AM, Coultas CW. Measuring Team Cohesion: Observations from the Science. Human Factors. 2015;57(3):365–374. https://doi.org/10.1177/0018720815578267.

[3] Bell ST, Brown SG, Colaneri A, Outland N. Team composition and the ABCs of teamwork. American Psychologist. 2018;73(4):349. https://doi.org/10.1037/amp0000305.

[4] Antone JQ, Mesmer-Magnus JR, Carter DR, DeChurch LA. Spaceflight Team Composition and Computational Modeling: Advancing Research to Enable Deep Space Exploration. Frontiers in Psychology. 2020;11:2115. https://doi.org/10.1201/9780429440878-6.

[5] Lungeanu A, Mesmer-Magnus JR, Niler AA, DeChurch LA, Contractor NS. Organizing for Mars: A Task Management Perspective on Work within Spaceflight Multiteam Systems. Human Factors. 2023;65(6):1199–1220. https://doi.org/10.1177/00187208221129939.

[6] Mesmer-Magnus JR, Carter DR, Asencio R, DeChurch LA. Space Exploration Illuminates the Next Frontier for Teams Research. Group & Organization Management. 2016;41(5):595–628. https://doi.org/10.1177/1059601116668763.

[7] Ilgen DR, Hollenbeck JR, Johnson M, Jundt D. Teams in organizations: From input–process–output models to IMOI models. Annual Review of Psychology. 2005;56(1):517–543. https://doi.org/10.1146/annurev.psych.56.091103.070250.

[8] Musson DM, Sandal GM, Helmreich RL. Personality characteristics and trait clusters in final stage astronaut selection. Aviation, Space, and Environmental Medicine. 2004;75(4):342–349.

[9] Kanas N, Sandal GM, Boyd JE, Gushin VI, Manzey D, North R, et al. Psychology and culture during long-duration space missions. Acta Astronautica. 2009;64(7-8):659–677. https://doi.org/10.1016/j.actaastro.2008.12.005.

[10] Landon LB, Rokholt C, Slack KJ, Pecena Y. Selecting astronauts for long-duration exploration missions: Considerations for team performance and functioning. REACH. 2017;5:33–56. https://doi.org/10.1016/j.reach.2017.03.002.

[11] Sandal GM, Bye HH, van de Vijver FJR. Personal values and crew compatibility: Results from a 105 days simulated space mission. Acta Astronautica. 2011;69(3-4):141–149. https://doi.org/10.1016/j.actaastro.2011.02.007.

[12] Bishop JW, Scott KD, Maynard-Patrick S, Wang L. Teams, Team Process, and Team Building. In: Garcia LS, Bachner P, Baselski VS, Lewis MR, Linscott AJ, Schwab DA, et al., editors. Clinical Laboratory Management. ASM Press; 2013. p. 373–391.
