# Supplementary material for: Exploring team dynamics and performance in extended space missions using agent-based modeling
Source: PLoS One. 2025 Oct 8;20(10):e0332496. doi: 10.1371/journal.pone.0332496 (PMC12507196; doi:10.1371/journal.pone.0332496)
Supplement: Attachment — Submitted filename: Response to Reviewers_PLOSONE_R2.pdf [file pone.0332496.s002.pdf]

Dear Editors and Reviewers,

Thank you for taking the time to review our manuscript again and for offering thoughtful feedback. Your comments have been very helpful in clarifying the aims of our study, strengthening the theoretical framing, and improving the overall clarity of our presentation.

In this revised version, we have carefully addressed all remaining points in a detailed, point-by-point response. We believe the changes further improve the rigor and readability of the manuscript, and we are grateful for the opportunity to refine the work in line with your suggestions.

Sincerely,

Iser Pena, Stevens Institute of Technology
Hao Chen, Stevens Institute of Technology

Reviewer 1

My previous comments #1-3 and #6-7 have all been sufficiently addressed. Re: previous comments #4-5. Thank you for the addition of the 2x2 design examining how both heterogeneity of personality composition and heterogeneity of skills/roles differentially predict stress, performance, and health in your model. I think disentangling these effects is important for enabling mission planners to effectively select and compose their teams for long-duration space missions. I can also tell you put a lot of work into the new analyses, results, and corresponding figures. However, because this was such a big overhaul of your design and results, there needs to be more work done to guide the reader through this aspect of your paper. As of right now, the 2x2 design appears abruptly in your paper without sufficient explanation as to why it is important to examine these two types of heterogeneity. I can think of two possible ways to address this issue, but perhaps you have other ideas that are even better: a) You could edit your abstract, intro, lit review, etc. to make the case for studying heterogeneity in two forms (personality and skills/roles), so that this extended analysis becomes a key contribution of your paper b) You could keep the current focus on personality heterogeneity but include the analyses with the 2x2 after including a discussion about why the heterogeneity of skills/roles is an important control variable in your model and then discuss whether or not personality heterogeneity sufficiently explained variance in your outcomes above and beyond that of skills/role heterogeneity.

We sincerely thank the reviewer for this thoughtful and constructive feedback. We agree that the 2×2 design required stronger integration across the paper and that its motivation needed to be clearer to the reader. Following the reviewer's suggestion, we revised the abstract, introduction, literature review, and Section 2.3 to explicitly position personality and skill/role heterogeneity as two central dimensions of team composition.

Page 1 (Abstract): "To capture functional as well as psychological diversity, the framework also incorporates variation in skills and roles, enabling a 2×2 factorial design that disentangles the effects of personality heterogeneity, role heterogeneity, and their interaction."

Page 1 (Abstract): "Our results suggest that team composition influences stress, health, performance, and cohesion, with personality variation and role specialization contributing distinctly. When combined, these factors appear to generate interaction effects relevant for crew selection, training, and preparation. These findings underscore the importance of jointly considering personality and functional diversity when developing strategies to support resilience and effectiveness in long-duration spaceflight."

Page 3 (Introduction): "Beyond individual resilience, effective mission functioning also depends on how skills and responsibilities are distributed across the crew. Evidence from spaceflight and analog environments suggests that redundant or overlapping roles can create inefficiencies, whereas complementary expertise helps sustain operations under isolation and delayed communication [7,9]. Analyses of multiteam systems further point to role differentiation and task management as essential coordination mechanisms when crews must operate with high autonomy [3]."

Page 3 (Introduction): "Despite this recognition, computational studies have rarely examined personality heterogeneity and functional specialization together. Most agent-based work isolates either personality variation or task-based diversity, leaving their combined influence underexplored [10].

Page 3 (Introduction):" This study addresses that need by formalizing both dimensions within a unified 2×2 factorial design, enabling direct comparison of their separate and interactive

contributions during simulated Mars-class missions. Our research introduces a novel ABM framework that incorporates the Five-Factor Model (FFM) of personality traits to simulate team dynamics during extended space missions."

Page 7 (Literature Review): "In addition to psychological factors, several studies point to the operational importance of skill diversity in spaceflight teams. For example, Sandal et al. [12] found in a 105-day space simulation that mismatches in functional responsibilities could create stress and inefficiencies, while balanced specialization improved perceived compatibility. Bishop et al. [13] likewise emphasized that clear differentiation of tasks and expertise supported stable performance in simulated long-duration crews. More broadly, reviews of team research in space and analog environments argue that composition must be understood across multiple dimensions, including both psychological compatibility and functional specialization [10]. However, most computational studies of spaceflight teams continue to model only one of these forms of heterogeneity at a time, leaving open questions about how personality diversity and role allocation interact in shaping stress, cohesion, and performance."

Pages 11–13, We completely updated Section 2.3 – Team Composition Conditions

Comments:

1. On page 2, you state that you are "explicitly modeling how psychological adaptation interacts with individual personality traits." What exactly does this mean? The way it is phrased, I would expect to see psychological adaptation be a key variable in your model, but it is not (I think it's more likely a potential explanatory mechanism). I would review and rephrase this to make sure it is clear what you mean.

   We appreciate this comment and recognize that our phrasing may have implied that "psychological adaptation" was introduced as a distinct variable within our model. In practice, we operationalize adaptation as the dynamic expression of stable personality traits under chronic stressors, influencing stress, performance, and cohesion outcomes over time. Thus, adaptation is not a separate equation or variable, but rather the explanatory lens through which we interpret how personality modulates behavioral responses. We have revised the introduction to clarify this distinction.

   Page 2: "Our study responds to this need by modeling how personality traits influence stress responses and resilience across long-duration missions. We interpret these dynamics as psychological adaptation, not as an isolated construct, but as the patterned ways in which traits shape coping and performance under chronic stress."

2. Your introduction to ABM on p. 6 comes very abruptly with no transition from the previous section. In your writing, you first need to make the case for ABM and its benefits for investigating these research questions before you introduce the technicalities of ABM in section 1.1.3.

   We agree with the reviewer's observation that our introduction to ABM appeared abruptly. To address this, we added a transition paragraph that first motivates the use of computational modeling and explains why ABM is well-suited for investigating astronaut team dynamics. We think this addition now provides a clearer bridge between the discussion of psychological and behavioral factors and the technical details of ABM.

   Page 7: "Traditional empirical and analog studies have provided valuable insights into

stress, adaptation, and personality in spaceflight, but they remain limited in scope. Most capture only short durations or isolated factors, making it difficult to study the nonlinear and cumulative dynamics of multi-year missions [6, 11]. Moreover, they cannot easily integrate individual heterogeneity (e.g., stable personality traits) and system-level processes (e.g., feedback loops between stress, cohesion, and performance). Computational modeling approaches provide a way to address these challenges by enabling controlled, repeatable experiments on complex social systems [2]. In particular, Agent-Based Modeling (ABM) offers a framework for linking micro-level variation among individuals to emergent team-level outcomes across time. Building on these limitations, the next section considers how ABM has been applied to study team dynamics and performance in spaceflight and other extreme environments."

3. You mention on P. 8 that ABMs can be used by mission planners to model diverse agents etc., but I highly doubt mission planners are loading up an ABM to test theses team composition questions. Instead, they likely review research (such as yours) that used an ABM along with their own knowledge to make decisions. Perhaps consider rephrasing (unless you know that they are definitely using ABMs like this to make their team composition decisions).

We thank the reviewer for this observation. The text has been revised to clarify that ABM is used as a research tool, with its results contributing indirectly to mission planning.

Page 9: "Among the available computational approaches, ABM has become a practical tool for examining how team processes unfold under spaceflight conditions. Rather than claiming to replace operational planning, these models offer structured experiments that reveal how differences in individual traits, coping styles, and interaction rules can accumulate into patterns at the team level. The value of this work lies in providing a systematic evidence base complementary to analog studies that helps mission planners and researchers anticipate potential risks and evaluate strategies to support crews. In this sense, ABM contributes not as a stand-alone decision system but as an analytic lens that extends what can be learned from empirical studies of long-duration missions [4, 5]."

4. Should Figure 1 say personal traits or personality traits? Why is cultural adaptation underlined?

Thank you for pointing this out. We revised Figure 1 for clarity. The label has been corrected to read *personality traits* rather than *personal traits*, and the underline on cultural adaptation has been removed to avoid unintended emphasis.

5. The role specialization portion of your paper (beginning in section 2.3) comes out of nowhere for a normal reader since they are not privy to these reviews. This is also not mentioned in your intro or abstract, or really anywhere else. However, the reason you have to separate out these effects is because your "story" was about how heterogeneity of teams impacts team success.

We sincerely thank the reviewer for this thoughtful and constructive feedback. We agree that the 2×2 design required stronger integration across the paper and that its motivation needed to be clearer to the reader. Following the reviewer's suggestion, we revised the abstract, introduction, literature review, and Section 2.3 to explicitly position personality and skill/role heterogeneity as two central dimensions of team composition.

Page 1 (Abstract): "To capture functional as well as psychological diversity, the framework also incorporates variation in skills and roles, enabling a 2×2 factorial design that disentangles the effects of personality heterogeneity, role heterogeneity, and their interaction."

Page 1 (Abstract): "Our results suggest that team composition influences stress, health, performance, and cohesion, with personality variation and role specialization contributing distinctly. When combined, these factors appear to generate interaction effects relevant for crew selection, training, and preparation. These findings underscore the importance of jointly considering personality and functional diversity when developing strategies to support resilience and effectiveness in long-duration spaceflight."

Page 3 (Introduction): "Beyond individual resilience, effective mission functioning also depends on how skills and responsibilities are distributed across the crew. Evidence from spaceflight and analog environments suggests that redundant or overlapping roles can create inefficiencies, whereas complementary expertise helps sustain operations under isolation and delayed communication [7, 9]. Analyses of multiteam systems further point to role differentiation and task management as essential coordination mechanisms when crews must operate with high autonomy [3]."

Page 3 (Introduction): "Despite this recognition, computational studies have rarely examined personality heterogeneity and functional specialization together. Most agent-based work isolates either personality variation or task-based diversity, leaving their combined influence underexplored [10].

Page 3 (Introduction):" This study addresses that need by formalizing both dimensions within a unified 2×2 factorial design, enabling direct comparison of their separate and interactive contributions during simulated Mars-class missions. Our research introduces a novel ABM framework that incorporates the Five-Factor Model (FFM) of personality traits to simulate team dynamics during extended space missions."

Page 7 (Literature Review): "In addition to psychological factors, several studies point to the operational importance of skill diversity in spaceflight teams. For example, Sandal et al. [12] found in a 105-day space simulation that mismatches in functional responsibilities could create stress and inefficiencies, while balanced specialization improved perceived compatibility. Bishop et al. [13] likewise emphasized that clear differentiation of tasks and expertise supported stable performance in simulated long-duration crews. More broadly, reviews of team research in space and analog environments argue that composition must be understood across multiple dimensions, including both psychological compatibility and functional specialization [10]. However, most computational studies of spaceflight teams continue to model only one of these forms of heterogeneity at a time, leaving open questions about how personality diversity and role allocation interact in shaping stress, cohesion, and performance."

Pages 11–13, We completely updated Section 2.3 – Team Composition Conditions

6. Your discussion of how skill heterogeneity is included in your model (bottom of page 12 and top of page 13) does not match your current 2x2 configuration. Please be sure to re-read your entire manuscript in light of the changes you have made to ensure that your descriptions still make sense, because right now these descriptions are not in alignment with this version of your model.

> We thank the reviewer for pointing out this inconsistency. In response, we carefully re-read the manuscript and revised the entire Section 2.3 (pp. 12–13). The updated section now fully aligns with the 2×2 factorial design Table 2, clarifying how both personality heterogeneity and skill/role heterogeneity are defined, operationalized, and combined into the four experimental conditions.
>
> Pages 12–13: We completely updated Section 2.3 "Team Composition Conditions"

7. In Figure 7, are you still showing "stress, performance, and health"? There are no labels for these in the figure.

> Thank you for pointing this out. To improve clarity, we have added explicit labels for "stress," "performance," and "health" in Figure 7, ensuring consistency with the labels.

8. This is a much improved version of your paper, and I hope these additional comments enable you to boost the quality of this work even more.

> We sincerely thank the reviewer for the encouraging assessment and for providing constructive suggestions. We carefully considered each point raised and have revised the manuscript accordingly. We believe these changes further strengthen the clarity, consistency, and contribution of the paper, and we are grateful for the reviewer's guidance in helping us refine this work.

References

[1] Bell ST, Brown SG, Abben DR, Outland NB. Team composition issues for future space exploration: A review and directions for future research. Aerospace Medicine and Human Performance. 2015;86(6):548–556. `https://doi.org/10.3357/AMHP.4251.2015`.

[2] Macal CM, North MJ. Tutorial on agent-based modelling and simulation. Journal of Simulation. 2010;4(3):151–162. `https://doi.org/10.1057/jos.2010.3`.

[3] Lungeanu A, Mesmer-Magnus JR, Niler AA, DeChurch LA, Contractor NS. Organizing for Mars: A Task Management Perspective on Work within Spaceflight Multiteam Systems. Human Factors. 2023;65(6):1199–1220. `https://doi.org/10.1177/00187208221129939`.

[4] Antone JQ, Mesmer-Magnus JR, Carter DR, DeChurch LA. Spaceflight Team Composition and Computational Modeling: Advancing Research to Enable Deep Space Exploration. Frontiers in Psychology. 2020;11:2115. `https://doi.org/10.1201/9780429440878-6`.

[5] Acquisti A, Sierhuis M, Clancey WJ, Bradshaw JM. Agent-based modeling of collaboration and work practices onboard the International Space Station. In: Proceedings of the Computer-Generated Forces and Behavior Representation Conference; 2002. DOI:10.2139/ssrn.4254197. Available from SSRN: `https://papers.ssrn.com/sol3/papers.cfm?abstract_id=4254197`

[6] Salas E, Grossman R, Hughes AM, Coultas CW. Measuring Team Cohesion: Observations from the Science. Human Factors. 2015;57(3):365–374. `https://doi.org/10.1177/0018720815578267`.

[7] Landon LB, Slack KJ, Barrett JD. Teamwork and collaboration in long-duration space missions: Going to extremes. American Psychologist. 2018;73(4):563–575. https://doi.org/10.1037/amp0000260.

[8] Mendl M. Performing under pressure: Stress and cognitive function. Applied Animal Behaviour Science. 1999;65(3):221–244. https://doi.org/10.1016/S0168-1591(99)00088-X.

[9] Bell ST, Brown SG, Mitchell T. What we know about team dynamics for long-distance space missions: A systematic review of analog research. Frontiers in Psychology. 2019;10:953. https://doi.org/10.3389/fpsyg.2019.00811.

[10] Mesmer-Magnus JR, Carter DR, Asencio R, DeChurch LA. Space Exploration Illuminates the Next Frontier for Teams Research. Group & Organization Management. 2016;41(5):595–628. https://doi.org/10.1177/1059601116668763.

[11] Kanas N, Sandal GM, Boyd JE, Gushin VI, Manzey D, North R, et al. Psychology and culture during long-duration space missions. Acta Astronautica. 2009;64(7-8):659–677. https://doi.org/10.1016/j.actaastro.2008.12.005.

[12] Sandal GM, Bye HH, van de Vijver FJR. Personal values and crew compatibility: Results from a 105 days simulated space mission. Acta Astronautica. 2011;69(3-4):141–149. https://doi.org/10.1016/j.actaastro.2011.02.007.

[13] Bishop JW, Scott KD, Maynard-Patrick S, Wang L. Teams, Team Process, and Team Building. In: Garcia LS, Bachner P, Baselski VS, Lewis MR, Linscott AJ, Schwab DA, et al., editors. Clinical Laboratory Management. ASM Press; 2013. p. 373–391.
